# The frequencies of *CYP2D6* alleles and their impact on clinical outcomes of adjuvant tamoxifen therapy in Syrian breast cancer patients

**DOI:** 10.1186/s12885-022-10148-8

**Published:** 2022-10-15

**Authors:** Wouroud Ismail Al-khalil, Lana Al-Salhi, Sara Rijjal, Majd Aljamali, Lama A. Youssef

**Affiliations:** 1 Department of Pharmaceutics and Pharmaceutical Technology, Program of Clinical and Hospital Pharmacy, Faculty of Pharmacy, Damascus University, Mezzeh Autostrad, Damascus, Syrian Arab Republic; 2 Department of Biochemistry and Microbiology, Faculty of Pharmacy, Damascus University, Damascus, Syrian Arab Republic; 3 Faculty of Pharmacy, International University for Science and Technology (IUST), Ghabagheb, Daraa Syrian Arab Republic; 4 National Commission for Biotechnology (NCBT), Damascus, Syrian Arab Republic

**Keywords:** Breast cancer, Tamoxifen, *CYP2D6*, Genotyping, CYP2D6*4, CYP2D6*10, CYP2D6*41, CYP2D6*69, Syria

## Abstract

**Background:**

Tamoxifen is one of the fundamental pillars of adjuvant endocrine therapy for hormone receptor-positive breast cancer; however, 30–50% of patients receiving tamoxifen experience tumor relapse. CYP2D6, encoded by an extremely polymorphic *CYP2D6* gene, is the rate-limiting enzyme of tamoxifen bioactivation. This study aimed at determining the frequencies of the most clinically relevant *CYP2D6* alleles and evaluating their impact on the responsiveness to tamoxifen in a cohort of Syrian breast cancer patients.

**Methods:**

This case–control study encompassed positive estrogen and/or progesterone receptor, stage 1–3 breast cancer female patients receiving tamoxifen at Al-Bairouni University Hospital, the major National Oncology Center in Syria. Successfully genotyped eligible patients (*n* = 97) were classified according to their response into; no recurrence group (*n* = 39) who had completed a five-year recurrence-free adjuvant tamoxifen therapy, and recurrence group (*n* = 58) who had experienced recurrence. Several star alleles including CYP2D6*4, CYP2D6*10, CYP2D6*41, and CYP2D6*69 were identified via targeted sequencing of specific polymerase chain reaction (PCR) products and phenotypes were assigned according to activity score (AS). The correlation between genotypes and disease-free survival (DFS) was assessed using Kaplan–Meier method and log-rank test. Hazard ratios were estimated using Cox proportional hazards regression models.

**Results:**

The allelic frequencies of CYP2D6*41, CYP2D6*10, CYP2D6*4, and CYP2D6*69 were found to be 9.28%, 7.22%, 7.22%, and 2.58%, respectively. No statistically significant differences were observed in the frequencies of *CYP2D6* phenotypes between the two arms (*P* = 0.24), nor the incidence of tamoxifen-induced hot flashes (*P* = 0.109). Poor metabolizers (PMs) tended to display shorter DFS than intermediate metabolizers (IMs) and normal metabolizers (NMs) combined (adjusted HR = 2.34, 95% CI = 0.84–6.55, *P* = 0.104). Notably, patients homozygous for the null CYP2D6*4 allele (1847A/A) had an elevated risk of disease recurrence compared to patients with 1847G/G genotype (adjusted HR = 5.23, 95% CI = 1.22–22.49, *P* = 0.026).

**Conclusions:**

Our findings show no association between *CYP2D6* phenotype and treatment outcomes of tamoxifen in Syrian breast cancer patients. Nevertheless, a worse DFS was revealed in patients with 1847A/A genotype (*4/*4).

**Supplementary Information:**

The online version contains supplementary material available at 10.1186/s12885-022-10148-8.

## Background

Female breast cancer has become the most commonly diagnosed cancer worldwide with nearly 2.3 million new cases according to GLOBOCAN 2020 estimates. Moreover, it remains the leading cause of global cancer mortality among female cancer patients [1]. Approximately, 75% of newly diagnosed breast cancers are hormone receptor-positive, and endocrine treatment represents the standard of care for these patients [2]. Five-year of adjuvant tamoxifen therapy has lowered the annual recurrence rate by almost a half and breast cancer mortality rate by approximately a third in both pre- and post-menopausal breast cancer patients [3].

The Achilles' heel of tamoxifen therapy is its being a prodrug with relatively weak anti-estrogenic properties. Tamoxifen requires extensive biotransformation by hepatic cytochrome P450 (CYP) enzymes to its two highly active metabolites; 4-hydroxy-tamoxifen (4-OH-TAM) and 4-hydroxy-N-desmethyl-tamoxifen (endoxifen). Efficacy is mainly attributed to endoxifen due to its higher serum concentrations (5–10 times) than that of 4-OH-TAM [4, 5].

CYP2D6 is the rate-limiting enzyme catalyzing tamoxifen bioactivation. It is encoded by a highly polymorphic *CYP2D6* gene with approximately 150 allelic variants identified to date [6]. *CYP2D6* alleles impose different enzymatic activity; for instance, they can be non-functional alleles (e.g., *3, *4, *5, and *6), reduced-function alleles (e.g., *10, *17, and *41), normal-function alleles (e.g., *1, *2, *33, and *35), or increased function alleles (*1xN, *2xN, and *35xN). *CYP2D6* genotype is later translated into four distinctive phenotypes based on each individual's combination of *CYP2D6* alleles: poor metabolizers (PMs), intermediate metabolizers (IMs), normal metabolizers (NMs), and ultrarapid metabolizers (UMs) [7].

Despite the remarkable efficacy of tamoxifen in the treatment of hormone receptor-positive breast cancer, a considerable percentage (30–50%) of patients on tamoxifen experience a relapse and eventually die of the disease [3]. It has been suggested that inherited *CYP2D6* genetic variations can explain 39–58% of the variability in the plasma concentrations of endoxifen [8]. Many prospective and retrospective studies have been conducted to investigate the association between *CYP2D6* genotype and tamoxifen efficacy, which led to notably conflicting results as reviewed by Mulder et al. (2021) [9].

Despite the multitude of genetic studies evaluating the frequencies of *CYP2D6* alleles worldwide, the genetic profile of *CYP2D6* in the Middle East populations is quite underestimated [10]. Moreover, pharmacogenetic studies assessing the influence of *CYP2D6* genotypes on tamoxifen efficacy in breast cancer patients from this region are rather scarce. Our study aimed at gaining insights into the frequencies of the most clinically relevant *CYP2D6* alleles in a cohort of Syrian breast cancer patients and establishing the clinical utility of *CYP2D6* genotyping in the prediction of tamoxifen efficacy and occurrence of its side effects.

## Materials and methods

### Study design and subjects

This observational case–control study was approved by the Scientific Research Bioethics Committee at the Faculty of Pharmacy, Damascus University, and the Institutional Review Board of Al-Bairouni University Hospital.

Inclusion criteria encompassed pre- and postmenopausal breast cancer patients aged 18 years or older with hormone receptor-positive breast cancer and had been treated with adjuvant tamoxifen therapy (20 mg per day). Patients were excluded if they had at least one of the following: a histologically confirmed diagnosis of both ER and PR (progesterone receptor) negative breast cancer, previous malignancies other than breast cancer, undergone other endocrine therapies (e.g., aromatase inhibitors and/or gonadotropin-releasing hormone agonists), received tamoxifen as palliative therapy in the metastatic settings, experienced a recurrence during the first three months of treatment initiation since the time required to reach the steady-state plasma concentrations of endoxifen is usually 2–3 months, and finally, discontinued treatment due to the occurrence of severe adverse effects such as endometrial hyperplasia and thromboembolic events. All enrolled patients gave written informed consents and were face-to-face interviewed at Al-Bairouni University Hospital, the largest oncology center in the nation that admits 70% of all cancer patients in Syria, between January and June of 2019 [11]. Collected demographic data and relevant information included age, menopausal status upon diagnosis, and side effects (i.e., hot flashes) of tamoxifen. Furthermore, information regarding the tumor characteristics, type of operative surgery, chemotherapy, radiotherapy, and date of the first relapse was collected from patients’ medical records.

Eligible patients were stratified according to the clinical outcomes of adjuvant tamoxifen therapy into two arms; a control arm that consisted of patients who had completed five-year treatment with tamoxifen without recording any sign of recurrence, and a case arm that included patients who had breast cancer recurrence.

### Genotyping

Peripheral blood samples were collected into EDTA tubes, and genomic DNA was extracted using a solution-based DNA isolation kit (Wizard® Genomic DNA Purification Kit, Promega®, USA) according to the manufacturer’s instructions. DNA samples were genotyped for CYP2D6*4, CYP2D6*10, and CYP2D6*41 alleles by standard sequencing of the specific polymerase chain reaction (PCR) amplicons containing the gene loci that encode their corresponding single nucleotide polymorphisms (SNPs) 1847G>A, 100C>T, and 2989G>A, respectively. Moreover, the reading lengths of the sequenced PCR products have enabled the screening for additional 24 variants and identifying 19 further star alleles of varying functions, where the variant is unique to the star allele (Additional File 1).

The PCR mixture was prepared using 50–100 ng of template gDNA, 20 μL of master mix (OnePCR™, GeneDirex®, Taiwan), and 0.5 μL of 10 pmol/μL of each specific primer, thereafter the volume was completed to 40 μL with nuclease-free water. PCR was performed in the Labcycler Basic (011–103)® (SensoQuest®, Germany) as previously described for each amplification with minor modifications to optimize conditions [12–14]. Primer pairs and PCR conditions are summarized in Additional Files 2 and 3, respectively. PCR amplicons were electrophoresed on an ethidium bromide stained 1.5% agarose gel and visualized under *ultraviolet* (UV) light.

For each of the three PCR reactions, a single band corresponding to the expected product length [355, 271, and 340 base pairs (bp)], indicated a successful amplification of the regions containing the targeted SNPs: 1847G>A, 100C>T, and 2989G>A, respectively. DNA sequencing was performed according to the standard protocols at Macrogen® Inc (Seoul, South Korea). Briefly, PCR products were purified by an enzymatic clean-up method using EnzSAP™ PCR Clean-up Reagent, and subsequently sequenced using the ABI PRISM® BigDye™ Terminator Cycle Sequencing Kit with an ABI PRISM® 3730XI DNA Analyzer. Chromas® 2.6.6 software (Technelysium Pty Ltd®, Australia) was used to view sequencing chromatograms.

### Allele assignment, activity scores, and phenotypes

Designation of star-alleles was based on *CYP2D6* haplotypes, which are defined as the specific combinations of SNPs identified during genetic analysis, as follows; CYP2D6*4 (100T, 1847A), CYP2D6*10 (100T, 1847G), CYP2D6*41 (100C, 2851T, 2989A), and CYP2D6*69 (100T, 2851T, 2989A) [6].

2851C>T exists on numerous star alleles of varying functions, and the normal function CYP2D6*2 allele is one of the most common alleles harboring this variant. CYP2D6*2 can be identified by genotyping two SNPs; 2851C>T and 4181G>C [6]. In our analysis the reading lengths did not allow the detection of the 4181G>C SNP; however, for genotyping purposes and due to the strong well established linkage disequilibrium between these two SNPs, patients whose haplotype harbors the 2851C>T SNP solely were assigned by default to carry the normal function CYP2D6*2 allele. CYP2D6*1 allele (alias the wild-type allele) was assigned in case any of the aforementioned variants was absent.

Phenotypes were defined by calculating the activity score (AS) according to Caudle et al. [7]. The non-functional alleles *4 and *69 were given a value of 0, and the decreased function alleles *10 and *41 were given values of 0.25 and 0.5, respectively, whereas a value of 1 was assigned to the normal function *1 and *2 alleles. The activity score was subsequently calculated by summing the activity values assigned to each allele. Patients were considered PMs if their AS was 0, IMs if their AS ranged from 0.25 to 1, and NMs if they had an AS between 1.25 and 2.25.

### Endpoint and statistical analyses

An independent-sample t-test was used to compare the means of continuous variables between the recurrence and the no recurrence groups. Chi-square test and Fisher’s exact test were applied for the comparison of categorical clinicopathologic characteristics and frequencies of *CYP2D6* alleles between the two groups. The observed frequencies of *CYP2D6* alleles were compared to the expected frequencies using Hardy–Weinberg equilibrium (HWE). Disease-free survival (DFS) was defined as the time from the initiation of therapy to the occurrence of first breast cancer event (local, regional, or distant/metastatic recurrence), the appearance of a new invasive contralateral breast cancer, the appearance of a second primary invasive cancer (non-breast cancer), or death from any cause [15].

The correlation between *CYP2D6* genotype and DFS was assessed using Kaplan–Meier method and log-rank test. Patients with no recurrence who had completed a five-year recurrence-free tamoxifen therapy were censored at the end of treatment. Univariate Cox proportional hazards analysis was used to identify significant factors associated with DFS and estimate hazard ratios (HRs) and their corresponding 95% confidence intervals (CIs). Consequently, significant variables were used as covariates in the multivariate analysis.

Statistical analyses were conducted using SPSS® (version 25) and GraphPad Prism® (version 8) software. A bilateral *P*-value less than 0.05 was considered to be statistically significant in all performed tests.

## Results

### Patients’ characteristics

**Fig. 1:**
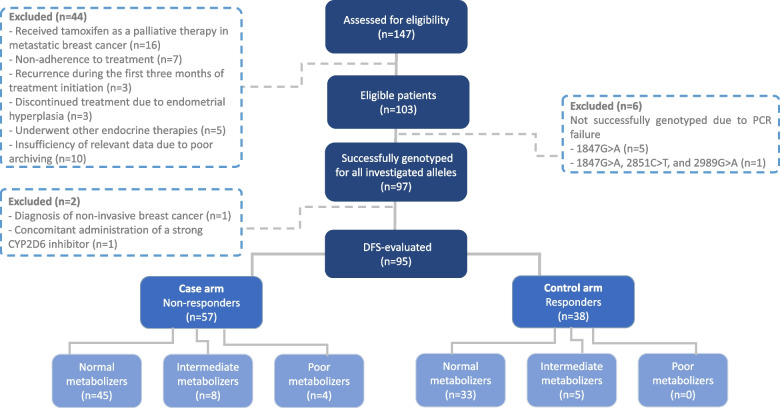
A flow diagram of participants and their study-arm allocation

A total of 103 breast cancer patients met the inclusion criteria; nonetheless, only 97 eligible patients were successfully genotyped for *CYP2D6*. Patients were classified according to their responsiveness to tamoxifen therapy into two arms; 58 patients with recurrence and 39 patients with no recurrence for five years. However, the impact of *CYP2D6* genotype on tamoxifen efficacy was evaluated in 95 patients as two patients were excluded; one patient presented with non-invasive breast cancer (Paget’s disease) and the other was concurrently on a strong CYP2D6 inhibitor (fluoxetine) as depicted in Fig. 1. Baseline clinical characteristics of the overall study population (*n* = 97) are listed in Table 1. The mean age was 48.81 years and more than half of the patients had reached menopause at the time of diagnosis (53.6%). The majority of the patients underwent mastectomy (91.8%) and received both adjuvant chemo- and radiotherapy (87.6%). Approximately 75% of the tumors were both ER + PR + , while human epidermal growth factor receptor 2 (HER2) positivity was evident in 20.6% of the patients. The overall patient characteristics were comparable between the two groups (*P* > 0.05). However, a higher percentage of regional lymph node involvement was observed in patients with recurrence (*P* = 0.03). Moreover, hot flashes were reported at a higher frequency (53.8%) among patients with no recurrence versus (32.8%) in patients with recurrence, but the difference did not reach statistical significance (*P* = 0.061).

**Table 1:** Baseline clinical characteristics of patients included in the study

Characteristics	Overall Patients (*n* = 97) N (%)	Recurrence (*n* = 58) N (%)	No recurrence (*n* = 39) N (%)	*P*-value
**Age at diagnosis (years)**				
Mean ± Standard deviation	48.81 ± 9.186	48.74 ± 9.484	48.92 ± 8.845	0.924
Range	26–68	30–67	26–68	
**Menstrual status**				
Premenopausal	30 (30.9)	19 (32.8)	11 (28.2)	0.812
Postmenopausal	52 (53.6)	31 (53.4)	21 (53.8)	
Perimenopausal	15 (15.5)	8 (13.8)	7 (17.9)	
**Hormonal receptors status**				
ER + PR +	72 (74.2)	47 (81)	25 (64.1)	0.08
ER + PR-	12 (12.4)	6 (10.3)	6 (15.4)	
ER-PR +	10 (10.3)	3 (5.2)	7 (17.9)	
Unknown	3 (3.1)	2 (3.4)	1 (2.6)	
**HER2 status**				
Positive	20 (20.6)	14 (24.1)	6 (15.4)	0.296
Negative	60 (61.9)	32 (55.2)	28 (71.8)	
Unknown	17 (17.5)	12 (20.7)	5 (12.8)	
**Nodal status**				
0	21 (21.6)	8 (13.8)	13 (33.3)	0.03
1–3	25 (25.8)	14 (24.1)	11 (28.2)	
≥ 4	46 (47.4)	33 (56.9)	13 (33.3)	
Unknown	5 (5.2)	3 (5.2)	2 (5.1)	
**Tumor size**				
Tis	1 (1)	0 (0)	1 (2.6)	0.387
T1	8 (8.2)	3 (5.2)	5 (12.8)	
T2	56 (57.7)	35 (60.3)	21 (53.8)	
T3	19 (19.6)	13 (22.4)	6 (15.4)	
T4	6 (6.2)	3 (5.2)	3 (7.7)	
Unknown	7 (7.2)	4 (6.9)	3 (7.7)	
**Operative surgery**				
Mastectomy	89 (91.8)	51 (87.9)	38 (97.4)	0.138
Breast-conserving	8 (8.2)	7 (12.1)	1 (2.6)	
**Other adjuvant therapies**				
Chemotherapy	11 (11.3)	7 (12.1)	4 (10.3)	0.459
Chemotherapy + Radiotherapy	85 (87.6)	51 (87.9)	34 (87.2)	
Neither	1 (1)	0 (0)	1 (2.6)	
**Hot flashes**				
Yes	40 (41.2)	19 (32.8)	21 (53.8)	0.061
No	55 (56.7)	37 (63.8)	18 (46.2)	
Unknown	2 (2.1)	2 (3.4)	0 (0)	

### The *CYP2D6* genotypes and predicted phenotype profiles

**Fig. 2:**
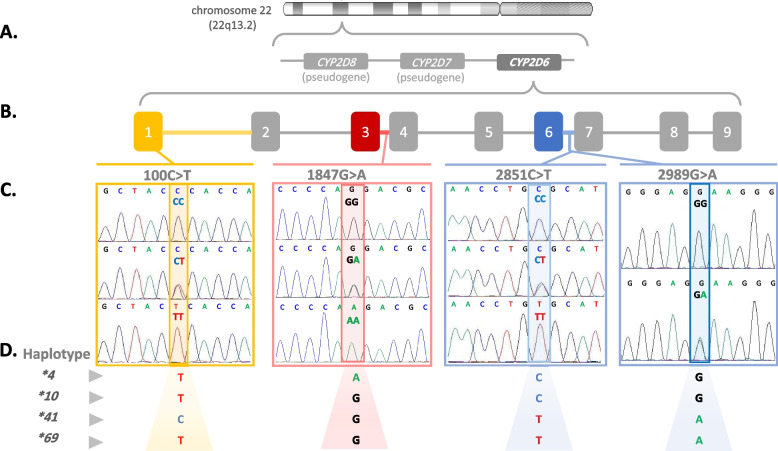
A schematic representation of *CYP2D6* gene cluster and DNA sequence chromatograms of the investigated variants. **A** *CYP2D6* gene and the two homologous *CYP2D7* and *CYP2D8* pseudogenes located on the long arm of chromosome 22 (22q13.2). **B** *CYP2D6* gene containing nine exons. 100C>T is located on the first exon (yellow box), 1847G>A is located on the third intron (red box), and 2851C>T as well as 2989G>A are located on the sixth intron (blue box). **C** Chromatograms in the upper line represent homozygosity of the wild type allele, the second line represent the heterozygosity and the lower line represent homozygosity of the variant allele. **D** *CYP2D6* inferred haplotypes according to the resultant sequencing chromatograms

A total of 27 variants and 23 star alleles were screened; however, only five SNPs (77G>A, 100C>T, 1847G>A, 2851C>T, and 2989G>A) and four-star alleles (CYP2D6*4, CYP2D6*10, CYP2D6*41, and CYP2D6*69) were identified in the study population (Fig. 2).

A detailed description of the observed frequencies of *CYP2D6* genotypes and their corresponding phenotypes is summarized in Table 2. The allelic frequencies of 77G>A and 2989G>A SNPs were consistent with HWE, whilst 100C>T, 1847G>A, and 2851C>T exhibited significant deviations from HWE (Additional File 4).

**Table 2:** Frequencies of *CYP2D6* genotypes and predicted phenotypes according to AS

Phenotypes	Genotypes	AS	N (%)
**NMs** **(1 < AS ≤ 2.25)**	*1/*1	2	22 (22.7)
	*1/*2	2	37 (38.1)
	*2/*2	2	1 (1)
	*1/*41	1.5	14 (14.4)
	*2/*41	1.5	3 (3.1)
	*1/*10	1.25	1 (1)
	*2/*10	1.25	1 (1)
**IMs** **(0 < AS ≤ 1)**	*1/*4	1	3 (3.1)
	*2/*4	1	1 (1)
	*41/*10	0.75	1 (1)
	*10/*10	0.5	2 (2.1)
	*10/*4	0.25	4 (4.1)
	*10/*69	0.25	3 (3.1)
**PMs** **(AS = 0)**	*4/*4	0	2 (2.1)
	*4/*69	0	2 (2.1)

### The association between *CYP2D6* genotype and clinicopathological characteristics

No correlation was found between *CYP2D6* genotype and tumor characteristics including hormonal receptors status, HER2 status, and tumor size. Nonetheless, approximately all patients (94.6%) carrying at least one function-altering variant (AS < 2) had shown an increased risk to have a lymph node-positive disease compared to patients whose haplotypes do not harbor any variant with deleterious effect on CYP2D6 activity (AS = 2) [odds ratio (OR) = 9.24, 95% CI = 2.25–41.72, *P* = 0.0009].

### Correlation between *CYP2D6* genotype and clinical outcomes

Although the four patients with the PM phenotype were all in the recurrence group, no statistically significant difference was noted regarding the frequency of different phenotypes between both groups (*P* = 0.24) (Table 3). Moreover, there was no statistically significant difference in hot flashes incidence between the three different phenotype groups (*P* = 0.109).

**Table 3:** Distribution of CYP2D6 phenotypes according to responsiveness and reported hot flashes

Phenotype	Recurrence (*n* = 57) N (%)	No recurrence (*n* = 38) N (%)	*P*-value	Hot flashes		*P*-value
				**Yes (*n* = 40) N (%)**	**No (*n* = 55) N (%)**	
**NM**	45 (57.7)	33 (42.3)	0.240	36 (46.2)	42 (53.8)	0.109
**IM**	8 (61.5)	5 (38.5)		2 (15.4)	11 (84.6)	
**PM**	4 (100)	0 (0)		2 (50)	2 (50)	

**Fig. 3:**
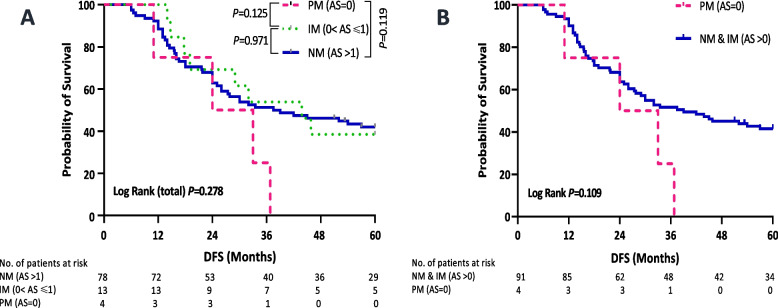
Kaplan–Meier estimates of DFS according to *CYP2D6* phenotypes in the successfully genotyped patients (*n* = 95). A. all metabolizer groups. B. PM vs*.* IM and NM combined

DFS was evaluated in each CYP2D6 phenotypic group. The results demonstrated that PMs had the shortest DFS of 28.5 months compared to IMs (43.8 months) and NMs (38.25 months), but this difference lacked statistical significance (*P* = 0.278, Fig. 3, A). However, PMs had a trend towards an increased risk of disease recurrence relative to IMs and NMs combined (HR = 2.24, 95% CI = 0.81–6.24, *P* = 0.123, Fig. 3, B). Nodal status was the only independent prognostic factor associated with DFS in the univariate analysis (number of positive nodes: 0–3 vs. ≥ 4, HR = 1.93, 95% CI = 1.12–3.35, *P* = 0.019). A multivariate analysis adjusted for nodal status did not influence the aforementioned observed trend between PM phenotype and DFS (adjusted HR = 2.34, 95% CI = 0.84–6.55, *P* = 0.104, Table 4).

**Table 4:** Univariate and multivariate analyses of DFS in tamoxifen-treated breast cancer patients

Variables		Univariate analysis			Multivariate analysis		
		**Unadjusted HR**	**95% CI**	***P*-value**	**Adjusted HR**^**a**^	**95% CI**	***P*-value**
***CYP2D6* phenotype**	**IM & NM**	1.00 (ref)	**-**	0.123	1.00 (ref)	**-**	0.104
	**PM**	2.24	0.81–6.24		2.34	0.84–6.55	
CYP2D6^*^4 genotype	**GG**	1.00 (ref)	**-**	**-**	1.00 (ref)	**-**	**-**
	**GA**	1.40	0.63–3.11	0.404	1.38	0.62–3.07	0.429
	**AA**	4.13	0.98–17.31	0.053	5.23	1.22–22.49	0.026

^a^HR was adjusted for nodal status (0–3 vs. ≥ 4)

**Fig. 4:**
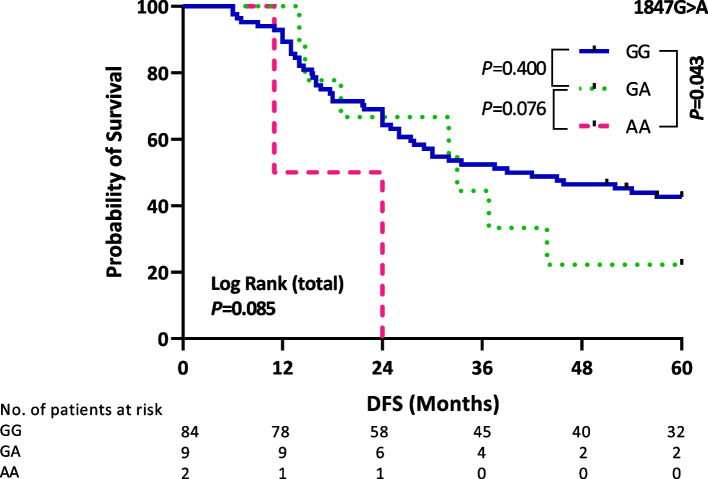
Kaplan–Meier estimates of DFS according to CYP2D6*4 genotype (1847G > A) in the successfully genotyped patients (*n* = 95)

DFS was also evaluated according to the genotype of each investigated SNP solely. There was no association between DFS and 2989G>A or 100C>T genotypes (*P* = 0.521 and *P* = 0.210, respectively, Additional File 5). Notably, patients who are homozygous for CYP2D6*4 null allele (1847A/A) displayed a significantly shorter DFS (17.5 months) than those with the 1847G/G genotype (40.5 months) (*P* = 0.043, Fig. 4). Furthermore, a multivariate analysis revealed a significant contribution of CYP2D6*4 homozygous genotype (1847A/A) to DFS (adjusted HR = 5.23, 95% CI = 1.22–22.49, *P* = 0.026, Table 4).

## Discussion

To the best of our knowledge, this is the first study that has identified the genetic profile of *CYP2D6* and evaluated the speculated association between *CYP2D6* genotype and clinical outcomes of adjuvant tamoxifen therapy in a cohort of Syrian breast cancer patients. Historically, the pivotal location of Syria has made its inhabitants extensively admixed with other populations through trade, immigration, wars, and marriages. This in turn resulted in a distinguished ethnic, cultural and genetic diversity.

*CYP2D6* polymorphisms were present in 78.4% of the assessed patients. Interestingly, we were able to identify CYP2D6*69 allele which carries the defining SNPs for CYP2D6*41 (2989G>A) and CYP2D6*10 (100C>T) in addition to others [16]. CYP2D6*69 is poorly identified in other populations. However, the observed frequency in our study (2.58%) was higher than that reported in Han Chinese (1.23%) by Qian et al. (2013) [17].

In our study, significant deviations from HWE for the 100C>T, 1847G>A, and 2851C>T SNPs were observed. These deviations can be most probably attributed to copy number variations, since gene deletion (CYP2D6*5) was not investigated in this study [18].

The previously reported robust correlation between *CYP2D6* genotype and endoxifen plasma concentrations has raised the hypothesis that patients with a normal capacity of CYP2D6 are expected to have superior outcomes when receiving tamoxifen. In the preceding few decades, the clinical utility of *CYP2D6* genotyping was a subject of immense interest as numerous studies have sought to test the *CYP2D6*/tamoxifen hypothesis. Nevertheless, given the limited and conflicting evidence, the clinical relevance of *CYP2D6* testing is yet to be established [9]. The Clinical Pharmacogenetics Implementation Consortium (CPIC) recommends genotype-based tamoxifen dosing or using an alternative hormonal therapy for both PMs and IM [19]. On the contrary, the National Comprehensive Cancer Network (NCCN) Breast Cancer Panel along with the American Society of Clinical Oncology (ASCO) and the European Society for Medical Oncology (ESMO) recommend against using *CYP2D6* genotype to guide the selection of adjuvant endocrine therapy [20–22]. Importantly, the FDA has asserted the influence of *CYP2D6* genotype on endoxifen concentrations, but not overall clinical outcomes [23]. Therefore, these discrepancies stress the importance of performing additional studies that support or refute the predictive value of *CYP2D6* genotyping prior to tamoxifen initiation.

Our present study failed to confirm the putative association between *CYP2D6* genotype and tamoxifen efficacy or occurrence of hot flashes. However, the effect of *CYP2D6* polymorphisms was more pronounced in PM patients, as a trend towards a shorter DFS with an elevated risk of disease recurrence was observed in PMs. Therefore, it is reasonable to postulate that although CYP2D6 activity is diminished in IMs, the metabolic capacity of the encoded enzyme is still capable of converting tamoxifen to its active form at adequate concentrations to competitively antagonize ERs in tumor breast cancer tissues. This is further supported by Hertz et al. (2016) who observed that endoxifen concentrations (7.10 ng/mL) among IMs exceed the putative threshold required for tamoxifen efficacy (5.9 ng/mL), whereas endoxifen concentrations are substantially below this threshold in PMs (3.4 ng/mL) [24]. However, the absence of a statistically significant correlation could be attributed to the small number of PMs (*n* = 4) identified in our genetic analysis [*4/*4 (*n* = 2) and *4/*69 (*n* = 2)].

Importantly, a sub-analysis excluding two PM patients with the *4/*69 in our study revealed that only one PM variant, CYP2D6*4 (1847G>A), was significantly associated with a decreased DFS as patients with homozygous CYP2D6*4 (A/A) genotype experienced a five-fold increased hazard to disease relapse compared to patients with G/G genotype. The CYP2D6*69 allele was first discovered as a novel haplotype by Gaedigk et al. (2009) in a patient phenotyped as a PM using dextromethorphan as a *CYP2D6* probe drug [16]. Further studies are needed to evaluate the consequences of CYP2D6*69 on CYP2D6 metabolic capacity using other substrates including tamoxifen.

Our results are consistent with those of a number of previous reports. In a retrospective study by Jorge-Aarón et al. (2020), there was no statistical difference found in the recurrence risk of breast cancer between the phenotypic groups (PM and IM) vs*.* (NM and UM) [25]. Similarly, Hertz et al. (2017) proved no association between *CYP2D6* genotype and tamoxifen efficacy [26]. Conversely, Damodaran et al. (2012) and Schroth et al. (2009) have demonstrated that reduced CYP2D6 activity in PM and IM patients was associated with poor response to tamoxifen and shorter recurrence-free survival [27, 28].

Our findings of a worse DFS in patients with the *4/*4 genotype compared to other genotypes are consistent with an earlier report by Goetz et al. (2005) [29]. Furthermore, our results are in line with that of Jansen et al. (2018) and Dezentje´et al. (2012) who found that the occurrence and severity of tamoxifen-induced hot flashes are not influenced by *CYP2D6* genotype nor endoxifen concentrations [30, 31]. On the contrary, a lower incidence of hot flashes was observed by Goetz et al. (2005) in patients with the *4/*4 genotype [29].

The strengths of this current study stem from the reliability of the genotyping method (amplicon sequencing) and DNA source, as well as its strict inclusion and exclusion criteria (non-adherent patients and those who received concomitant CYP2D6 inhibitors). However, due to the highly polymorphic nature of the gene encoding CYP2D6 and the multitude of allelic variants (over 140), a major limitation of our study is the relatively limited alleles identified (23 star alleles), which may lead to a faulty assignment of the wild-type allele in patients carrying deleterious SNPs, and consequently some misclassification of patients’ phenotypes. Furthermore, we did not assess the UM phenotype which is mostly predominant among Arabs (9.2%) [10]. Duplication events are observed for numerous alleles, thus doubling the value assigned for the allele being duplicated. This can considerably influence the assignment of an individual’s phenotype [7, 32]. However, apart from *CYP2D6* gene duplications, it is worth mentioning that we have genotyped the most prevalent and clinically significant *CYP2D6* alleles among Middle Easterners. We have also screened for the presence of some scarce alleles that could considerably affect CYP2D6 activity. A few other limitations include the relatively small sample size and the retrospective nature of the study that could have led to the loss of some important data and the potential of recall bias regarding the incidence of tamoxifen-associated side effects.

Intriguingly, breast cancer patients who carried at least one function-altering allele exhibited a significantly elevated risk of nodal metastases. However, we cannot currently drive any reasonable hypothesis to interpret the underlying mechanism and we cannot exclude the possibility of coincidental association.

Our findings demonstrate a lack of association between *CYP2D6* phenotype and clinical outcomes in Syrian breast cancer patients receiving adjuvant tamoxifen therapy. Future prospective studies are needed to determine the frequency of other *CYP2D6* alleles and copy number variations of this highly polymorphic gene in the Syrian population and investigate their potential impact on the response to tamoxifen hormonal therapy in breast cancer patients.

## Supplementary Information

**Additional file 1: Table S1.** Additional investigated variants and star alleles throughout sequencing.

**Additional file 2:** **Table S2.** Primers’ characteristics for PCR amplification of CYP2D6 gene locus containing the identified SNPs.

**Additional file 3: Table S3.** PCR conditions and steps for the identification of each SNP.

**Additional file 4:** **Table S4.** Frequencies of the identified SNPs, genotype data and results of Hardy-Weinberg Equilibrium (HWE).

**Additional file 5:** **Figure S1.** Kaplan-Meier estimates of DFS according to CYP2D6 genotype in the successfully genotyped patients (n=95). A. based on 2989G>A genotype solely. B. based on 100C>T genotype solely.

## Data Availability

The datasets used and/or analyzed during the current study are available from the corresponding author on reasonable request.

## Abbreviations

AS: Activity score
ASCO: The American Society of Clinical Oncology
bp: Base pair
CIs: Confidence intervals
CPIC: The Clinical Pharmacogenetics Implementation Consortium
CYP: Cytochrome P450
DFS: Disease-free survival
ER: Estrogen receptor
ESMO: The European Society for Medical Oncology
FDA: The Food and Drug Administration
HER2: Human epidermal growth factor receptor 2
HRs: Hazard ratios
HWE: Hardy–Weinberg equilibrium
IMs: Intermediate metabolizers
NCCN: The National Comprehensive Cancer Network
NMs: Normal metabolizers
4-OH-TAM: 4-Hydroxy-tamoxifen
OR: Odds ratio
PCR: Polymerase chain reaction
PMs: Poor metabolizers
PR: Progesterone receptor
SERM: Selective estrogen receptor modulator
SNPs: Single nucleotide polymorphisms
UMs: Ultrarapid metabolizers
UV: *Ultraviolet*
