# Supplementary material for: The frequencies of CYP2D6 alleles and their impact on clinical outcomes of adjuvant tamoxifen therapy in Syrian breast cancer patients
Source: BMC Cancer. 2022 Oct 15;22:1067. doi: 10.1186/s12885-022-10148-8 (PMC9571463; doi:10.1186/s12885-022-10148-8)
Supplement: Supplementary file 1 — Additional file 1: Table S1. Additional investigated variants and star alleles throughout sequencing. [file 12885_2022_10148_MOESM1_ESM.docx]

| **Table S1. Additional investigated variants and star alleles throughout sequencing** | | | |
| --- | --- | --- | --- |
| **PCR products** | **Variants** | **Star alleles** | **Function** |
| **100C>T-containing PCR product** | 73C>T | **47*^✛^ | No function |
|  | 77G>A | **43* | Uncertain function |
|  |  | **46* | Normal function |
|  |  | **146* | Not assigned |
|  | 82C>T | **22* | Uncertain function |
|  |  | **44* | No function |
|  |  | **124* | Not assigned |
|  | 124G>A | **12*^✛^ | No function |
|  | 125G>A | **71*^✛^ | Uncertain function |
|  | 137_138insT | **15*^✛^ | No function |
| **1847G>A-containing PCR product** | 1944G>A | **37*^✛^ | Uncertain function |
|  | 1913T>C | **130*^✛^ | Uncertain function |
|  | 1914G>T |  |  |
|  | 1977_1978insG | **20*^✛^ | No function |
|  | 1980T>C |  |  |
|  | 1864_1865ins  TTTCGCCCCTTTCGCCCC | **40*^✛^ | No function |
|  | 1864_1865insTTTCGCCCC | **58*^✛^ | Unknown function |
|  | 1888_1889insTA | **60*^✛^ | No function |
|  | 1996delC | **92*^✛^ | No function |
| **2989G>A-containing PCR product** | 2829delC | **100*^✛^ | No function |
|  | 2851C>T | **2* and 53 other alleles and sub alleles | Function varies according to the haplotype |
|  | 2854A>C | **24*^✛^ | Uncertain function |
|  | 2870T>C | **123*^✛^ | Unknown function |
|  | 2893A>G | **118*^✛^ | Unknown function |
|  | 2928delGATCCTACATCCGGATGTG | **101*^✛^ | No function |
|  | 2936A>C | **7*^✛^ | No function |
|  | 2940G>A | **59*^✛^ | Decreased function |
|  | 2951G>C | **44*^✛^ | No function |
| ^✛^A total of 19 additional star alleles were investigated (the variant is unique to the star allele) | | | |
