# Supplementary material for: The frequencies of CYP2D6 alleles and their impact on clinical outcomes of adjuvant tamoxifen therapy in Syrian breast cancer patients
Source: BMC Cancer. 2022 Oct 15;22:1067. doi: 10.1186/s12885-022-10148-8 (PMC9571463; doi:10.1186/s12885-022-10148-8)
Supplement: Supplementary file 2 — Additional file 2: Table S2. Primers’ characteristics for PCR amplification of CYP2D6 gene locus containing the identified SNPs. [file 12885_2022_10148_MOESM2_ESM.docx]

| **Table S2. Primers’ characteristics for PCR amplification of *CYP2D6* gene locus containing the identified SNPs** | | | | |
| --- | --- | --- | --- | --- |
| **Allele** | **Identifying SNP** | **Primers’ Sequences** | **Product Size (bp)** | **Reference** |
| ***CYP2D6*4*** | 1847G>A | For: 5'-GCCTTCGCCAACCACTCCG-3' | 355 | (14) |
|  |  | Rev: 5'-AAATCCTGCTCTTCCGAGGC-3' |  |  |
| ***CYP2D6*10*** | 100C>T | For: 5'-CCATTTGGTAGTGAGGCAGGTAT-3' | 271 | (12) |
|  |  | Rev: 5'-CACCATCCATGTTTGCTTCTGGT-3' |  |  |
| ***CYP2D6*41*** | 2989G>A | For: 5'-CCGTTCTGTCCCGAGTATGC-3' | 340 | (13) |
|  |  | Rev: 5'-CGGCCCTGACACTCCTTCTT-3' |  |  |
| For: forward, Rev: reverse | | | | |
