# Supplementary material for: The frequencies of CYP2D6 alleles and their impact on clinical outcomes of adjuvant tamoxifen therapy in Syrian breast cancer patients
Source: BMC Cancer. 2022 Oct 15;22:1067. doi: 10.1186/s12885-022-10148-8 (PMC9571463; doi:10.1186/s12885-022-10148-8)
Supplement: Supplementary file 3 — Additional file 3: Table S3. PCR conditions and steps for the identification of each SNP. [file 12885_2022_10148_MOESM3_ESM.docx]

| **Table S3. PCR conditions and steps for the identification of each SNP** | | | | | | |
| --- | --- | --- | --- | --- | --- | --- |
| **SNP** | **PCR conditions** | **Initial denaturation** | **Denaturation** | **Annealing** | **Elongation** | **Final elongation** |
| **100C>T** | Temp (⁰c) | 95 | 94 | 60 | 72 | 72 |
|  | Duration | 15 min | 30 sec | 45 sec | 45 sec | 10 min |
|  | Cycles |  | 35 cycles | | |  |
| **1847G>A** | Temp (⁰c) | 95 | 95 | 65 | 72 | 72 |
|  | Duration | 5 min | 30 sec | 30 sec | 1 min | 5 min |
|  | Cycles |  | 35 cycles | | |  |
| **2989G>A** | Temp (⁰c) | 95 | 95 | 55 | 72 | 72 |
|  | Duration | 5 min | 30 sec | 30 sec | 1 min | 10 min |
|  | Cycles |  | 32 cycles | | |  |
