# Supplementary material for: The frequencies of CYP2D6 alleles and their impact on clinical outcomes of adjuvant tamoxifen therapy in Syrian breast cancer patients
Source: BMC Cancer. 2022 Oct 15;22:1067. doi: 10.1186/s12885-022-10148-8 (PMC9571463; doi:10.1186/s12885-022-10148-8)
Supplement: Supplementary file 4 — Additional file 4: Table S4. Frequencies of the identified SNPs, genotype data and results of Hardy-Weinberg Equilibrium (HWE). [file 12885_2022_10148_MOESM4_ESM.docx]

| **Table S4. Frequencies of the identified SNPs, genotype data and results of Hardy-Weinberg Equilibrium (HWE)** | | | | | | | | | | | |
| --- | --- | --- | --- | --- | --- | --- | --- | --- | --- | --- | --- |
| **SNP** | **%** | **Genotype** | **Overall patients**  **(n=103)** | | | **Recurrence**  **(n=61)** | | | **No recurrence**  **(n=42)** | | |
|  |  |  | **Observed**  **N (%)** | **Expected**  **N** | **HWE**  ***P*-value** | **Observed**  **N (%)** | **Expected**  **N** | **HWE**  ***P*-value** | **Observed**  **N (%)** | **Expected**  **N** | **HWE**  ***P*-value** |
| **77G>A** | 0.5 | GG | 102 (99) | 102 | 0.961 | 61 (100) | 61 | N/A | 41 (97.6) | 41 | 0.938 |
|  |  | GA | 1 (1) | 1 |  | 0 (0) | 0 |  | 1 (2.4) | 1 |  |
|  |  | AA | 0 (0) | 0 |  | 0 (0) | 0 |  | 0 (0) | 0 |  |
|  |  | Missing | 0 (0) |  |  | 0 (0) |  |  | 0 |  |  |
| **100C>T** | 18.4 | CC | 78 (75.7) | 68.5 | **<0.0001** | 45 (73.8) | 39.4 | **<0.0001** | 33 (78.6) | 29.2 | **<0.0001** |
|  |  | CT | 12 (11.7) | 31 |  | 8 (13.1) | 13.9 |  | 4 (9.5) | 11.7 |  |
|  |  | TT | 13 (12.6) | 3.5 |  | 8 (13.1) | 2.4 |  | 5 (11.9) | 1.2 |  |
|  |  | Missing | 0 (0) |  |  | 0 (0) |  |  | 0 (0) |  |  |
| **1847G>A** | 7.22 | GG | 85 (87.6) | 83.5 | **0.023** | 49 (84.5) | 47.5 | **0.024** | 36 (92.3) | 36.1 | 0.803 |
|  |  | GA | 10 (10.3) | 13 |  | 7 (12.1) | 10 |  | 3 (7.7) | 2.9 |  |
|  |  | AA | 2 (2.1) | 0.5 |  | 2 (3.4) | 0.5 |  | 0 (0) | 0.1 |  |
|  |  | Missing | 6 (5.8) |  |  | 3 (4.9) |  |  | 3 (7.1) |  |  |
| **2851C>T** | 40.7 | CC | 26 (25.5) | 35.9 | **<0.0001** | 15 (25) | 20.4 | **0.004** | 11 (26.2) | 15.5 | **0.004** |
|  |  | CT | 69 (67.6) | 49.2 |  | 40 (66.7) | 29.2 |  | 29 (69) | 20 |  |
|  |  | TT | 7 (6.9) | 16.9 |  | 5 (8.3) | 10.4 |  | 2 (4.8) | 6.5 |  |
|  |  | Missing | 1 (1) |  |  | 1 (1.6) |  |  | 0 (0) |  |  |
| **2989G>A** | 11.27 | GG | 79 (77.5) | 80.3 | 0.199 | 47 (78.3) | 47.7 | 0.347 | 32 (76.2) | 32.6 | 0.381 |
|  |  | GA | 23 (22.5) | 20.4 |  | 13 (21.7) | 11.6 |  | 10 (23.8) | 8.8 |  |
|  |  | AA | 0 (0) | 1.3 |  | 0 (0) | 0.7 |  | 0 (0) | 0.6 |  |
|  |  | Missing | 1 (1) |  |  | 1 (1.6) |  |  | 0 (0) |  |  |
| N/A: Not applicable | | | | | | | | | | | |
