# Supplementary material for: The frequencies of CYP2D6 alleles and their impact on clinical outcomes of adjuvant tamoxifen therapy in Syrian breast cancer patients
Source: BMC Cancer. 2022 Oct 15;22:1067. doi: 10.1186/s12885-022-10148-8 (PMC9571463; doi:10.1186/s12885-022-10148-8)
Supplement: Supplementary file 5 — Additional file 5: Figure S1. Kaplan-Meier estimates of DFS according to CYP2D6 genotype in the successfully genotyped patients (n=95). A. based on 2989G>A genotype solely. B. based on 100C>T genotype solely. [file 12885_2022_10148_MOESM5_ESM.docx]

**A**

**Figure S1. Kaplan-Meier estimates of DFS according to *CYP2D6* genotype in the successfully genotyped patients (n=95). A. based on 2989G>A genotype solely. B. based on 100C>T genotype solely**

**B**
